# A highly efficient rice green tissue protoplast system for transient gene expression and studying light/chloroplast-related processes

**DOI:** 10.1186/1746-4811-7-30

**Published:** 2011-09-30

**Authors:** Yang Zhang, Jianbin Su, Shan Duan, Ying Ao, Jinran Dai, Jun Liu, Peng Wang, Yuge Li, Bing Liu, Dongru Feng, Jinfa Wang, Hongbin Wang

**Affiliations:** 1 State Key Laboratory of Biocontrol, School of Life Sciences, Sun Yat-sen University, Guangzhou 510275, P. R. China; 2 Key Laboratory of Gene Engineering of Ministry of Education, School of Life Sciences, Sun Yat-sen University, Guangzhou 510275, P. R. China; 3 Guangdong Key Laboratory of Plant Resources, School of Life Sciences, Sun Yat-sen University, Guangzhou 510275, P. R. China

## Abstract

**Background:**

Plant protoplasts, a proven physiological and versatile cell system, are widely used in high-throughput analysis and functional characterization of genes. Green protoplasts have been successfully used in investigations of plant signal transduction pathways related to hormones, metabolites and environmental challenges. In rice, protoplasts are commonly prepared from suspension cultured cells or etiolated seedlings, but only a few studies have explored the use of protoplasts from rice green tissue.

**Results:**

Here, we report a simplified method for isolating protoplasts from normally cultivated young rice green tissue without the need for unnecessary chemicals and a vacuum device. Transfections of the generated protoplasts with plasmids of a wide range of sizes (4.5-13 kb) and co-transfections with multiple plasmids achieved impressively high efficiencies and allowed evaluations by 1) protein immunoblotting analysis, 2) subcellular localization assays, and 3) protein-protein interaction analysis by bimolecular fluorescence complementation (BiFC) and firefly luciferase complementation (FLC). Importantly, the rice green tissue protoplasts were photosynthetically active and sensitive to the retrograde plastid signaling inducer norflurazon (NF). Transient expression of the GFP-tagged light-related transcription factor OsGLK1 markedly upregulated transcript levels of the endogeneous photosynthetic genes *OsLhcb1*, *OsLhcp*, *GADPH *and *RbcS*, which were reduced to some extent by NF treatment in the rice green tissue protoplasts.

**Conclusions:**

We show here a simplified and highly efficient transient gene expression system using photosynthetically active rice green tissue protoplasts and its broad applications in protein immunoblot, localization and protein-protein interaction assays. These rice green tissue protoplasts will be particularly useful in studies of light/chloroplast-related processes.

## Background

Transient expression assays allow rapid and high-throughput analysis of genes in plants [1,2] and thus have become widely used for characterization of gene function.* Arabidopsis*, maize [3] and tobacco protoplasts [4], tobacco leaf epidermal cells [5], tobacco BY-2 cells [6] and onion epidermal cells [7] are commonly used for transient assays in gene expression, protein subcellular localization, protein-protein interaction and protein activity studies. Accordingly, several methods for transient gene expression have been developed, such as PEG-mediated protoplast transfection [8], biolistic bombardment [9] and *Agrobacterium*-mediated transient transformation [10].

Rice is one of the most important cereal crops and a model species for monocotyledonous plants [11]. Some systems such as tobacco and onion have been used for characterization of rice genes [5-7], but they are heterologous systems; the expressed proteins in heterologous systems may exhibit aberrant traits. For example, the encoded proteins of some *Arabidopsis *genes introduced in tobacco have been shown to be mis-localized [2]. Therefore, many studies have attempted to establish efficient gene expression systems in rice, including tissue-based and individual cell-based methods. In tissue-based methods, rice calli, leaves and seedlings are used for transient assays by different approaches. The bombardment approach was successfully used to introduce DNA into rice calli and intact seedlings grown in the dark, but it had poor efficiency and depended on expensive equipment [12,13]. Similarly, an electroporation-mediated approach in rice leaves also showed low efficiency [14]. The *Agrobacterium-*mediated approach yielded higher efficiency and is inexpensive [15-17], but it is difficult to use for subcellular localization and other fluorescence-based analysis, as this method is often associated with a high level of non-specific autofluorescence. Moreover, the waxy structure of rice tissue is difficult to observe under a fluorescence microscope.

The other type of transient gene expression method used in rice is based on individual cells, including protoplasts and suspension cultured cells [18,19]. Green protoplasts provide a suitable system for the quantitative study of many physiological and biochemical processes of plant cells [20], especially light/chloroplast-related processes such as light-induced chloroplasts movement in tobacco [21,22] and light-regulated gene expression in maize [23]. However, suspension cultured cells and etiolated protoplasts are mainly used in transient gene expression assays currently in rice [18,19,24,25]. Suspension cultured cells and etiolated protoplasts cultured in the dark are not suitable for investigating many cellular processes, particularly those involving chloroplasts. Some efforts has been made to develop a protoplast transient gene expression system using rice green tissues, which has been used for developmentally regulated plant defense-related gene expression analysis [24], siRNA-mediated silencing [25] and subcellular localization assays [26]. Until now, however, there have been no reported studies of light/chloroplast-related processes using the protoplast system in rice.

Here, we present a simplified and highly efficient method for transient gene expression in protoplasts using young rice green tissue. We applied this method to express one or more constructs for protein immunoblotting, localization and protein-protein interactions assays, particularly for studies of light/chloroplast-related processes.

## Results

### Isolation of protoplasts from rice green tissue

**Figure 1:**
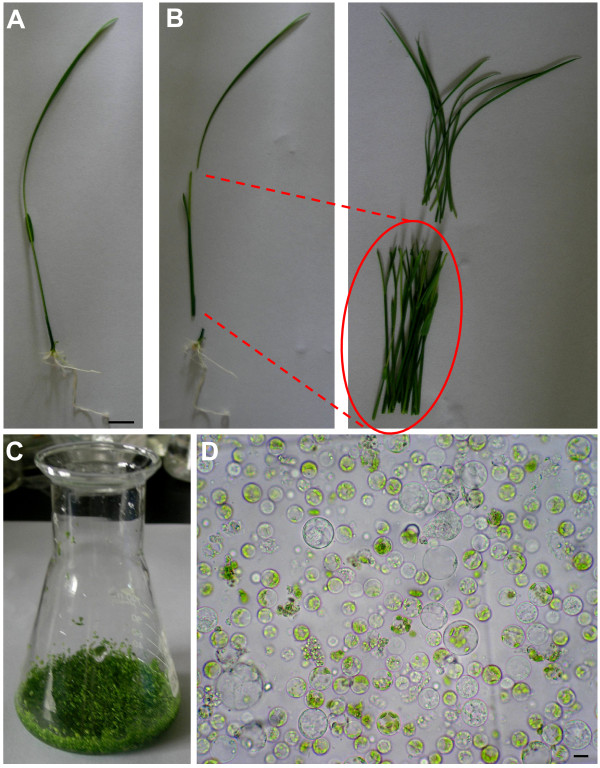
**Isolation of protoplasts from rice green tissue**. A, A representative healthy 8-day-old rice seedling used for protoplast isolation. Scale bar = 1 cm. B, Red markers indicate the optimal sections of seedlings (stem and sheath) yielding protoplasts. C, Cut strips were treated with 0.6 M mannitol followed by enzymatic digestion. D, Image of protoplasts obtained using a Nikon digital camera under an Olympus microscope with a 40× objective. Scale bar = 10 μm.

To establish a more physiological and versatile protoplast system than that of suspension cultured cells or etiolated seedlings, we chose normally cultured rice green seedlings as the source material. Briefly, 7 to 10-day-old rice green seedlings cultured at 26°C on 1/2 MS medium with a 12 h light (~150 μmol m^-2 ^s^-1^)/12 h dark cycle, were used for protoplast isolation (Figure 1A and Additional file 1). Stem and sheath tissues from 40-60 rice seedlings were cut into approximately 0.5 mm strips (Figure 1B). The strips were immediately transferred into 0.6 M mannitol for a quick plasmolysis treatment, followed by enzymatic digestion in the dark with gentle shaking (Figure 1C). The protoplasts were collected by filtration through 40 μm nylon meshes. In this isolation protocol, the use of toxic reagents, antibiotics and vacuum was not required.

**Figure 2:**
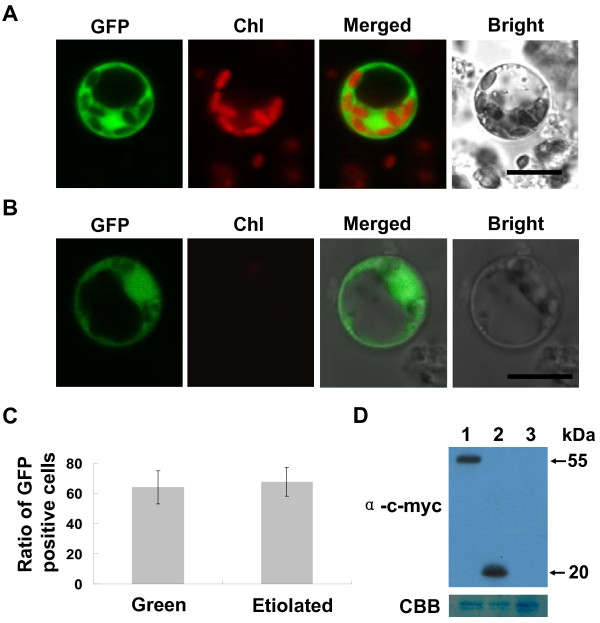
**Comparison of transfection efficiencies between rice green tissue protoplasts and etiolated protoplasts**. Transient expression of different constructs in rice protoplasts. Samples were visualized under a confocal microscope. A-B, The *35S::GFP *construct pUC-GFP was transiently expressed in protoplasts derived from 8-day-old rice green seedlings (A) or 8-day-old etiolated rice seedlings (B). Individual and merged images of GFP and chlorophyll autofluorescence (Chl) as well as bright field images of protoplasts are shown. Scale bars = 10 μm. C, The transfection efficiency of rice green tissue protoplasts was comparable to that of etiolated protoplasts. The ratio of GFP-positive cells to the total number of protoplasts (n ≥ 100) was scored as the transfection efficiency. Values are means, with standard errors indicated by bars, representing at least 5 replicates. D, Detection of transiently expressed bZIP63-c-myc-YFP^N ^(lane 1) and c-myc-YFP^N ^(lane 2) by Western blot. Lane 3 was a negative control. The upper panel shows an immunoblot using a monoclonal mouse anti-c-myc antibody; the lower panel shows a Coomassie Brilliant Blue (CBB)-stained PVDF membrane after immunoblotting as a loading control.

**Figure 3:**
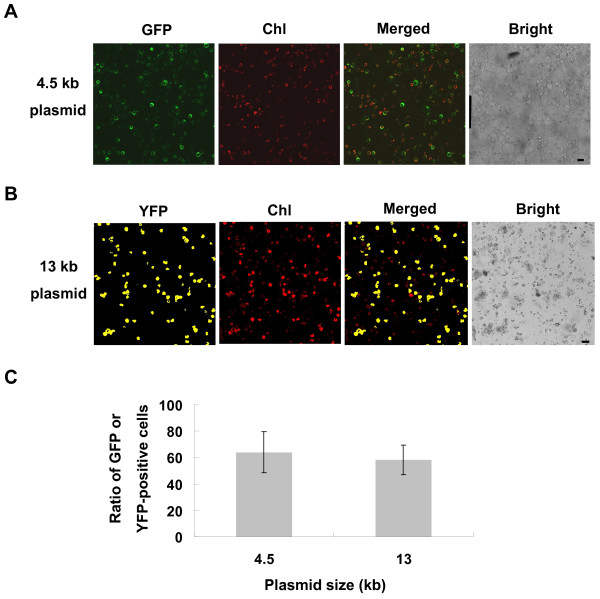
**Transient expression efficiencies of different sized plasmids in rice green tissue protoplasts**. A-B, A 4.5 kb plasmid pUC-GFP (A) and a 13 kb plasmid CD3-998 (B) were transiently expressed in rice green tissue protoplasts. Merged images of GFP or YFP and chlorophyll autofluorescence (Chl) as well as bright field images of protoplasts are shown. Scale bars = 20 μm. C, Transfection efficiency of a 13 kb plasmid compared with that of a 4.5 kb plasmid, expressed as the ratio of GFP or YFP-positive cells to the total number of protoplasts (n ≥ 100). Values are means, with standard errors indicated by bars, representing at least 5 replicates.

**Figure 4:**
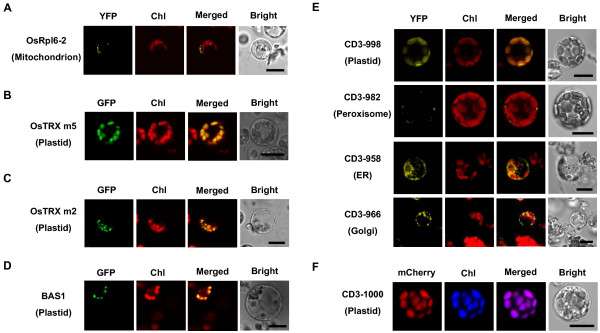
**Subcellular localization analysis in rice green tissue protoplasts**. Rice proteins and *Arabidopsis *organelle markers were transiently expressed in rice green tissue protoplasts. A, Rice OsRpl6-2-YFP labeling of mitochondria. B, Rice OsTRX m5-GFP labeling of plastids. C, OsTRX m2-GFP targeted to chloroplasts. D, BAS1-GFP targeted to chloroplasts. E, CD3-998 labeling of plastids; CD3-982 labeling of peroxisomes; CD3-958 labeling of the endoplasmic reticulum (ER); CD3-966 labeling of Golgi. F, An mCherry-based plastid marker CD3-1000. Merged images are shown with YFP, mCherry or GFP and chlorophyll autofluorescence (Chl). Bright field images of protoplasts are also shown. Scale bars = 10 μm.

**Figure 5:**
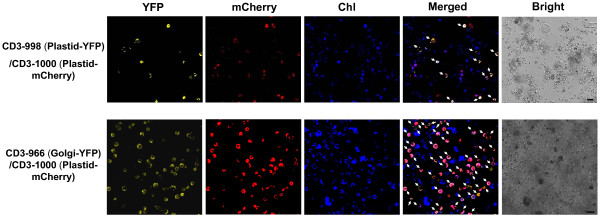
**Co-localization analysis with known organelle markers**. A, Plastid-YFP and plastid-mCherry markers, or B, Golgi-YFP and plastid-mCherry markers were co-expressed in rice green tissue protoplasts. Cells showing both markers in each co-transfection are indicated by arrowheads in the merged images of YFP, mCherry and chlorophyll autofluorescence (Chl) signals. Bright field images of protoplasts are also shown. Scale bar = 20 μm.

**Figure 6:**
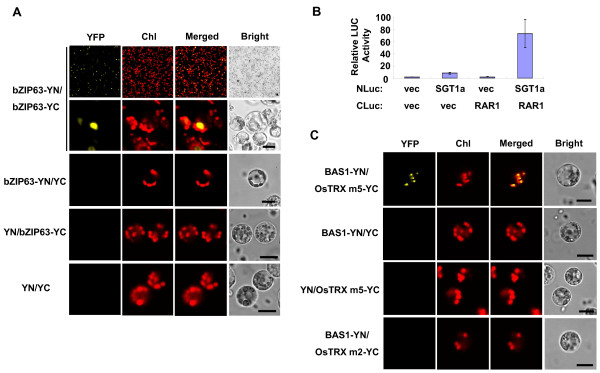
**Protein-protein interaction assays in rice green tissue protoplasts**. A, Protein-protein interaction analysis by BiFC. Construct pairs of pUC-bZIP63-YN (bZIP63-YN) and pUC-bZIP63-YC (bZIP63-YC), bZIP63-YN and pUC-SPYCE (YC), pUC-SPYNE (YN) and bZIP63-YC, or YN and YC were transiently co-expressed in rice green tissue protoplasts. BiFC fluorescence is indicated by the YFP signal. Individual and merged images of YFP and chlorophyll autofluorescence (Chl) as well as bright field images of protoplasts are shown. The upper panel contains low power images showing high co-expression efficiency. Scale bars = 10 μm. B, Protein-protein interaction analysis by FLC. Constructs as indicated plus the RNL construct as an internal control were transiently co-expressed in rice green tissue protoplasts. Firefly luciferase activity was normalized to RNL activity. Values are means, with standard errors indicated by bars, representing 3 replicates. C, Construct pairs of pUC-BAS1-YN (BAS1-YN) and pUC-OsTRX m5-YC (OsTRX m5-YC), BAS1-YN and YC, YN and OsTRX m5-YC, or BAS1-YN and pUC-OsTRX m2-YC (OsTRX m2-YC) were transiently co-expressed in rice green tissue protoplasts. BiFC fluorescence is indicated by the YFP signal. Individual and merged images of YFP and chlorophyll autofluorescence (Chl) as well as bright field images of protoplasts are shown. Scale bars = 10 μm.

Our method generated approximately 1 × 10^7 ^cells varying from 7 to 25 μm in size (Figure 1D), which was sufficient for more than 50 transfection experiments (2 × 10^5 ^cells per transfection). The generated rice green tissue protoplasts were above 95% viable judged by fluorescein diacetate (FDA) staining (Additional file 2). In the rice green tissue protoplasts, the chloroplasts could be easily identified by their typical chlorophyll autofluorescence under a confocal microscope (Chl channels of Figure 2, 3, 4, 5 and 6), while they could not be clearly observed in etiolated protoplasts (Figure 2B).

### Highly efficient transient transfection of different sized constructs in rice green tissue protoplasts

After transfection with the *35S::GFP *plasmid (pUC-GFP) by using the PEG-mediated transfection approach and incubation for 10 h, the GFP fluorescence was clearly detected both in the cytoplasm and nucleus of the rice green tissue protoplasts and etiolated protoplasts (Figure 2A and 2B). Transfection efficiencies of 53-75% were achieved in the rice green tissue protoplasts, comparable to the 58-77% in etiolated protoplasts (Figure 2C and 3A). Moreover, 45-66% transfection efficiencies were obtained using a large sized 13 kb binary plasmid CD3-998 in the rice green tissue protoplasts (Figure 3B and 3C). Even the co-expression of two 13 kb plasmids (CD3-998 and CD3-1000, or CD3-966 and CD3-1000) could be easily detected in one random visual field under a confocal microscope with maximum co-transfection efficiencies of 30-45% (Figure 5). Furthermore, the transfection efficiency was not significantly affected by the transfected plasmid DNA amount in the range of 5-15 μg (data not shown).

To test whether the amount of transiently expressed proteins could be detected in protein assays (e.g., Western blot), we transfected the pUC-bZIP63-YN and pUC-SPYNE constructs into 2 × 10^6 ^rice green tissue protoplast cells. The expressed proteins bZIP63-c-myc-YFP^N ^and c-myc-YFP^N ^at around 55 kDa and 20 kDa, respectively, were clearly detected by monoclonal mouse antibodies to c-myc (Figure 2D). The yield of proteins from 2 × 10^6 ^cells was sufficient for at least 10 immunoblot experiments, again indicating that the transfection efficiency was sufficient for protein assays.

### Subcellular localization studies in rice green tissue protoplasts

Four rice proteins, OsRpl6-2, OsTRX m5, OsTRX m2 and BAS1 were expressed as GFP fusion proteins in the rice green tissue protoplast system for subcellular localization studies. As the N-terminal coding region of rice OsRpl6-2 contains mitochondrial targeting information [27], we observed that OsRpl6-2-YFP was distributed in the cytosol of rice protoplasts as small fluorescent spots resembling mitochondria (Figure 4A). Rice OsTRX m5 (Ostrxm) is a chloroplast m type thioredoxin [28], and OsTRX m5-GFP appeared to co-localize with the red autofluorescence of chloroplasts (Figure 4B). OsTRX m2 was predicted to be a chloroplast m type thioredoxin as well (WoLFPSORT, http://wolfpsort.org/; TargetP 1.1 server, http://www.cbs.dtu.dk/services/TargetP/), and OsTRX m2-GFP located to the chloroplasts, exhibiting small fluorescent spots (Figure 4C). BAS1 is a rice chloroplastic 2-Cys peroxiredoxin [29], and BAS1-GFP presented distinctly as one fluorescent spot per chloroplast (Figure 4D). The demonstration of rice proteins targeting to the correct organelles indicated that the rice green tissue protoplast system is suitable for subcellular localization assays.

A set of heterologous *Arabidopsis *organelle markers was also tested for subcellular localization studies in this rice green tissue protoplast system (Additional file 3). The majority of the *Arabidopsis *organelle markers tested could target to the correct compartments. The YFP or mCherry fluorescence of *Arabidopsis *plastid marker proteins merged perfectly with the chlorophyll autofluorescence (Figure 4E and 4F), labeling chloroplasts of cells from green tissue. The *Arabidopsis *peroxisomes were observed as small and round organelles, exhibiting small fluorescent spots in association with the chloroplasts (Figure 4E). The *Arabidopsis *endoplasmic reticulum (ER) presented as an extensive network throughout the cytoplasm (Figure 4E). The *Arabidopsis *Golgi marker was observed as small, nearly round spots (Figure 4E). However, a few heterologous *Arabidopsis *markers showed ambiguous localization or partial mis-localization in the rice system. For example, some *Arabidopsis *ER markers produced an altered labeling pattern that surrounded the chloroplasts in a half-moon shape; and instead of labeling the plasma membrane (PM), the *Arabidopsis *PM marker localized to the cytosol and nucleus (Additional file 4).

Furthermore, we used fluorescently-tagged organelle markers to mimic co-localization analysis as co-localization with known organelle markers is often used to determine the location of a protein [2]. The plastid-YFP marker CD3-998 and plastid-mCherry marker CD3-1000 were found co-localized in the same chloroplasts (Figure 5, upper panel). Likewise, the co-expressed Golgi-YFP marker CD3-966 and plastid-mCherry marker CD3-1000 could easily be detected in the same cells (Figure 5, lower panel).

### Detection of protein-protein interactions in rice green tissue protoplasts

We further applied this method to investigate protein-protein interactions by bimolecular fluorescence complementation (BiFC) [30] and firefly luciferase complementation (FLC) assays [31]. The bZIP transcription factors are known to form homodimers in the nucleus [32]. Co-expression of the bZIP63-c-myc-YFP^N ^and bZIP63-HA-YFP^C ^fusion proteins in rice green tissue protoplasts produced obvious YFP signals in the nucleus (Figure 6A), consistent with previous results reported in *Arabidopsis *protoplasts and tobacco leaves [30]. As negative controls, co-expression of pUC-bZIP63-YN and empty pUC-SPYCE vectors, empty pUC-SPYNE and pUC-bZIP63-YC vectors, or two empty vectors pUC-SPYNE and pUC-SPYCE did not produce BiFC fluorescence (Figure 6A). The low power images in Figure 6A (upper panel) again demonstrated that a high co-transfection efficiency could be achieved in the rice green tissue protoplast system.

FLC assays were performed by using the SGT1a-NLuc and CLuc-RAR1 constructs, which carried the N-terminal and C-terminal halves of the luciferase protein, respectively. When co-expressed in cells, these constructs were expected to come together due to the known interaction of SGT1a with RAR1 [31], at the same time reconstituting a functional luciferase enzyme. A renilla luciferase (RNL) vector was co-transfected in all FLC experiments, serving as an internal control, and the firefly luciferase activity was normalized to RNL activity. As negative controls, co-transfections of the SGT1a-NLuc and empty 35S::CLuc vectors, the empty 35S::NLuc and CLuc-RAR1 vectors, or two empty vectors 35S::NLuc and 35S::CLuc, plus a RNL vector, did not show or exhibited only low background relative luciferase activity (between 2-8 units, Figure 6B). Meanwhile, co-transfection of the SGT1a-NLuc and CLuc-RAR1 constructs resulted in strong relative Luciferase activity (73 units), indicating a specific interaction (Figure 6B).

As a proof of concept, we used the rice green tissue protoplast system to detect whether OsTRX m5 and BAS1 interacted *in vivo*, as 2-Cys peroxiredoxin was suggested to be a potential target of thioredoxin OsTRX m5 [28]. Co-expression of the BAS1-c-myc-YFP^N ^and OsTRX m5-HA-YFP^C ^fusion proteins in rice green tissue protoplasts produced obvious YFP signals in the chloroplasts (Figure 6C), consistent with the subcellular location of BAS1 (Figure 4D). Meanwhile, OsTRX m2 did not interact with BAS1 in our experiment, and co-transfection of pUC-BAS1-YN and empty pUC-SPYCE vectors or empty pUC-SPYNE and pUC-OsTRX m5-YC vectors did not show BiFC fluorescence (negative controls, Figure 6C). These results further supported BAS1 as a potential target of thioredoxin OsTRX m5.

### Studies of light/chloroplast-related processes in rice green tissue protoplasts

**Figure 7:**
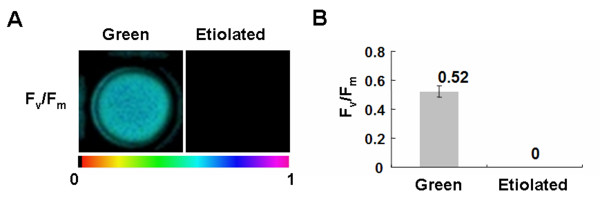
**Photosynthesis activivity of rice green tissue protoplasts**. A, Color images of the maximal PS II quantum yield (F_v_/F_m_) of rice green tissue and etiolated protoplasts. The false color ranged from black (0) via red, orange, yellow, green, blue and violet to purple (1) as indicated at the bottom. B, F_v_/F_m _in rice green tissue and etiolated protoplasts. Values are means, with standard errors indicated by bars, representing 7 replicates.

**Figure 8:**
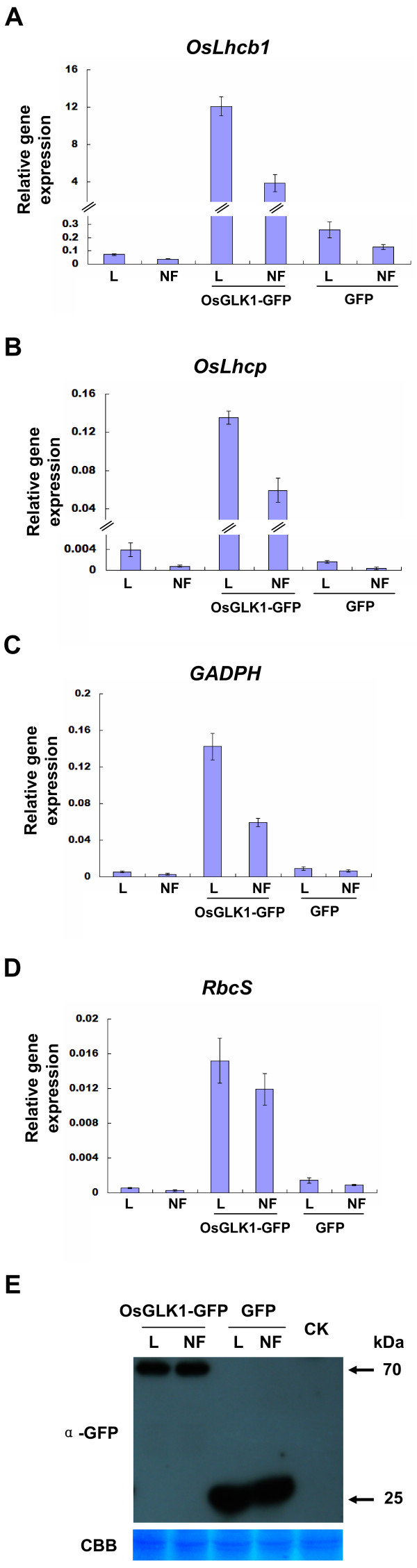
**Expression levels of OsGLK1-upregulated genes in rice green tissue protoplasts under light and NF treatments**. Protoplasts transfected with/without OsGLK1-GFP or GFP were treated with 40 μmol m^-2 ^s^-1 ^light and/or 500 nM NF for 12 h. A-D, Transcript levels of *OsLhcb1*, *OsLhcp*, *GADPH *and *RbcS *were detected by quantitative real-time PCR and normalized to that of *β-actin*. Values are means, with standard errors indicated by bars, representing 3 independent biological samples, each with 3 technical replicates. E, Detection of transiently expressed OsGLK1-GFP (lanes 1-2) and GFP (lanes 3-4) by Western blot. CK was a negative control. The upper panel shows an immunoblot using a monoclonal rabbit anti-GFP antibody. As a loading control, a Coomassie Brilliant Blue (CBB)-stained PVDF membrane is shown in the lower panel.

Rice green tissue protoplasts provide a physiological and versatile cell system to characterize gene functions, which may be potentially used to investigate light/chloroplast-related cellular processes. Thus, we first examined whether the rice green tissue protoplasts were photosynthetically active using an Imaging-PAM chlorophyll fluorometer. The imaging color of the maximum photosystem II quantum yield (F_v_/F_m_) [33] in etiolated protoplasts was black (F_v_/F_m _= 0), but that in rice green tissue protoplasts was light blue (F_v_/F_m _= 0.52), indicating the rice green tissue protoplast cells were photosynthetically active (Figure 7A and 7B). When rice green tissue protoplasts were treated with low light (40 μmol m^-2 ^s^-1^) and/or the retrograde plastid signaling inducer norflurazon (NF, 500 nM), the expressions of photosynthetic genes *OsLhcb1 *(chlorophyll a/b-binding protein 1), *OsLhcp *(LHCII type I CAB-2), *GADPH *(glyceraldehyde-3-phosphate dehydrogenase) and *RbcS *(nuclear-encoded small subunit of ribulose-1,5-bisphosphate carboxylase/oxygenase) were detected by quantitative real-time PCR. The transcript levels of the four genes in rice green tissue protoplasts were decreased by 20-55% under NF treatment (Figure 8A-D), indicating the rice green tissue protoplasts were sensitive to the retrograde plastid signaling inducer.

The results above suggested these rice protoplasts can be used for investigating light/chloroplast-related cellular processes. To further support this concept, we expressed a light-related transcription factor OsGLK1 combined with light and/or NF treatment in the rice green tissue protoplasts. GLK transcription factors are known to markedly upregulate the transcript levels of photosynthetic genes under light, while the transcript levels of both GLKs and photosynthetic genes are regulated by feedback signals from the plastid [34,35]. In our study, the four photosynthetic genes (*OsLhcb1*, *OsLhcp*, *GADPH *and *RbcS*) were upregulated by 30-168 fold in protoplasts expressing OsGLK1-GFP compared to those without OsGLK1-GFP expression, while NF treatment decreased the levels of these up-regulated genes by 30-75% (Figure 8A-D). Similarly, downward trends of transcript levels of *OsLhcb1*, *OsLhcp*, *GADPH *and *RbcS *(23-72%) were seen in the protoplasts expressing GFP with NF treatment compared with those expressing GFP only without NF treatment (Figure 8A-D). As a control, expression of the proteins OsGLK1-GFP (around 70 kDa) and GFP (27 kDa) were evaluated by Western blot using a monoclonal rabbit antibody to GFP, which clearly showed similar protein expressions in light and/or NF treatment (Figure 8E).

## Discussion

Although the rice green tissue protoplast system has been widely used in many applications, it does have a few limitations. For example, protoplasts cannot be used to characterize proteins localized to the cell wall or to study direct interactions between cells, since by definition individual protoplasts lack a cell wall and connections to other cells. However, as the results are shown above and discussed below, rice protoplasts from green tissue confer many advantages for plant biological studies. Here, we used rice green tissue to establish a physiological and versatile protoplast system for transient gene expression. We simplified the protoplast isolation protocol and systematically applied the rice green tissue protoplasts in protein immunoblot, localization and protein-protein interaction assays. Finally, we validated the use of the rice green tissue protoplasts in studies of light/chloroplast-related processes.

### A rapid and highly efficient transient gene expression system in rice green tissue protoplasts

The rice protoplast isolation method was simplified by removing use of unnecessary chemicals and a vacuum device from the protocol. The green stem and sheath of young rice green seedlings cultured at 26°C on 1/2 MS medium with a photoperiod of 12 h light (about 150 μmol m^-2 ^s^-1^)/12 h dark cycle were used as the source material. A short plasmolysis treatment before enzymatic digestion was used for osmoticum equilibrium, in order to maintain protoplast viability and reduce spontaneous protoplast fusion [36]. We found that fresh and tender rice seedlings were key to isolating protoplasts that could be transfected at high efficiencies, with 7 to 10-day-old seedlings being the most suitable for this purpose. The transfection efficiencies were variable in protoplasts from 11 to 14-day-old seedlings and declined sharply with those from seedlings older than 14 days. In contrast, other reported methods utilize 2-week-old or 2-month-old rice green tissue, antibiotics, toxic chemicals or vacuum [24,25].

Following PEG-mediated transfection [8], we achieved a maximum transfection efficiency of 75% using protoplasts isolated from rice green tissue (Figure 2C and 3A), matching that of the most effective transient gene expression system in etiolated rice protoplasts (70%) [24,25]. Maximum efficiencies of 45-66% were obtained with a large sized (13 kb) binary plasmid (Figure 3). Moreover, good transfection efficiencies were also obtained when two 13 kb plasmids (Figure 5) or three constructs (Figure 6B) were co-transfected. These results demonstrated that transfection using our rice green tissue protoplast system is a simple, highly efficient and rapid process, suitable for co-expressing multiple constructs of a wide range of sizes (4.5-13 kb).

### Suitability of rice green tissue protoplasts for analysis by protein immunoblotting, subcellular localization and protein-protein interactions assays

Transiently expressed proteins are often used in immunoblotting and activity assays [37]. In our study, the amount of protein expressed from a small scale transfection (5μg plasmid per 2 × 10^5 ^rice green tissue protoplast cells) was sufficient for protein assays (Figure 2D and 8E). As the transfection efficiency was not significantly different between 5-15 μg of plasmid DNA (data not shown), it stands to reason that probably using less DNA in the lower limit of this range with a higher number of protoplasts in a single transfection would increase total levels of expressed proteins to suit particular experimental needs.

Transient expression is also commonly applied in fluorescent-based assays, especially of target proteins fused to fluorescent tags for subcellular localization analysis. In our study, all rice plastid [28,29] and mitochondrion [27] fusion proteins targeted to their corresponding compartments in the rice green tissue protoplasts as expected (Figure 4A-D). Although the *Arabidopsis *plastids, peroxisomes, ER and Golgi markers [38] could largely target to their corresponding compartments in rice green tissue protoplast system as well (Figure 4E and 4F), we observed partial mis-localization of a few heterologous *Arabidopsis *markers in this system (Additional file 4). These findings suggested that it is always better to use a homologus system to study protein localization since a heterologous system may result in mis-targeting [2].

Proteins typically interact dynamically and form functional complexes to participate in signaling pathways and regulatory networks [39,40]. Thus, the ability to identify, examine and visualize protein-protein interactions in living cells is highly valuable. We were able to successfully apply the rice green tissue protoplast system to studying protein-protein interactions by using BiFC (Figure 6A and 6C), one of the most powerful tools for such analysis *in situ *using living cells [30,41]. The high co-transfection efficiency in rice green tissue protoplasts would no doubt facilitate the gathering of fluorescence data from large numbers of cells when performing BiFC analyses.

We also successfully used the rice green tissue protoplasts to perform the FLC assay, which was recently developed to quantitatively measure dynamic changes in protein-protein interactions in living cells [31,42]. The SGT1a and RAR1 interaction [31] was demonstrated to be 9-35 fold higher than the signal obtained with negative controls (Figure 6B), indicating the rice green tissue protoplasts can be used for dynamic protein interaction studies.

As a proof of concept, we also investigated subcellular localization and protein-protein interactions of OsTRX m2, OsTRX m5 and BAS1, which all targeted to the chloroplasts as predicted when expressed in the rice green tissue protoplast system, although with different patterns: OsTRX m2 as small fluorescent spots, OsTRX m5 filling the chloroplasts [28], and BAS1 as one large fluorescent spot per chloroplast (Figure 4B-D). These different localization patterns suggested specific localization of each protein inside chloroplasts. Moreover, we confirmed the interaction of BAS1 with OsTRX m5 *in vivo *(Figure 6C), further supporting BAS1 is a potential target of thioredoxin OsTRX m5 as previously suggested [28].

### Potential of rice green tissue protoplasts for studies of chloroplast or light-related cellular processes

As mentioned above, green protoplasts provide a suitable system for the study of many physiological and biochemical processes of plant cells [20]. The transcriptional activator Dof1 involved in light-regulated gene expression is activated in green protoplasts but not in etiolated protoplasts [23], suggesting that green protoplasts are more suitable in light/chloroplast-related studies as has been demonstrated in tobacco leaf and maize [21,22]. However, no such studies have been reported using rice protoplasts, currently obtained mainly from suspension cultured cells and etiolated cells [18,19,24,25] that are grown in the dark and lack light-dependent proteins or structures [43].

In this work, we demonstrated that the rice green tissue protoplasts were photosynthetically active (Figure 7) and sensitive to the retrograde plastid signaling inducer NF (Figure 8A-D), suggesting their potential to be used in light/chloroplast-related studies. The feasibility of such studies was further demonstrated when we showed that transient expression of a light-related transcription factor OsGLK1 markedly upregulated the transcript levels of various endogenous photosynthetic genes (*OsLhcb1*, *OsLhcp*, *GADPH *and *RbcS*), which were reduced to some extent by treatment with the retrograde plastid signaling inducer NF in rice green tissue protoplasts (Figure 8) [34,35]. It is conceivable that such experiments can be extended in future studies using the rice green tissue protoplast system for high-throughput gene analysis as has been demonstrated with various other plant protoplast systems [1].

## Conclusions

In conclusion, we show here that a physiological and versatile protoplast system, using fresh and tender rice green tissue, allowed for rapid and highly efficient DNA transfection for analysis by protein immunoblot, localization and protein-protein interaction assays. This system was successfully used for the simultaneous expression of multiple constructs and plasmids of a wide range of sizes. Notably, the protoplasts from rice green tissue, unlike those from etiolated or cultured suspension cells currently used, were demonstrated to be a useful system for studies of light/chloroplast-related processes.

## Methods

### Protoplast isolation

Dehulled seeds of rice (*Oryza sativa L*.) cultivar Nipponbare were sterilized with 75% ethanol for 1 min. These seeds were further sterilized with 2.5% sodium hypochlorite for 20 min, washed at least five times with sterile water and then incubated on 1/2 MS medium with a photoperiod of 12 h light (about 150 μmol m^-2 ^s^-1^) and 12 h dark at 26°C for 7-10 days. Green tissues from the stem and sheath of 40-60 rice seedlings were used. A bundle of rice plants (about 30 seedlings) were cut together into approximately 0.5 mm strips with propulsive force using sharp razors. The strips were immediately transferred into 0.6 M mannitol for 10 min in the dark. After discarding the mannitol, the strips were incubated in an enzyme solution (1.5% Cellulase RS, 0.75% Macerozyme R-10, 0.6 M mannitol, 10 mM MES at pH 5.7, 10 mM CaCl_2 _and 0.1% BSA) for 4-5 h in the dark with gentle shaking (60-80 rpm). After the enzymatic digestion, an equal volume of W5 solution (154 mM NaCl, 125 mM CaCl_2_, 5 mM KCl and 2 mM MES at pH 5.7) was added, followed by vigorous shaking by hand for 10 sec. Protoplasts were released by filtering through 40 μm nylon meshes into round bottom tubes with 3-5 washes of the strips using W5 solution. The pellets were collected by centrifugation at 1,500 rpm for 3 min with a swinging bucket. After washing once with W5 solution, the pellets were then resuspended in MMG solution (0.4 M mannitol, 15 mM MgCl_2 _and 4 mM MES at pH 5.7) at a concentration of 2 × 10^6 ^cells mL^-1^, determined by using a hematocytometer. The viability of protoplasts was determined by the FDA staining method as described [44]. All manipulations above were performed at room temperature.

For isolating protoplasts from etiolated rice seedlings, the sterilized seeds were germinated under light for 3 days, and then moved to the dark for another 4-7 days. The isolation procedure was the same as that for isolation of green tissue protoplasts described above.

### Plasmids

The recombinant plasmids used in this study are listed in Additional file 3. Plasmids pUC-GFP and pUC-YFP were derived from pUC 19 [45]. *OsTRX m2 *(Os04g0530600), *OsTRX m5 *(Os12g0188700), *BAS1 *(Os02g0537700) with introduced *Xba*I and *Xho*I sites, and *OsGLK1 *(AK098909) with introduced *Xba*I and *Spe*I sites were cloned from rice cDNA without the stop codon and inserted into pUC-GFP. The N-terminal coding region of *OsRpl6-2 *(Os08g0484301, 1-52 amino acids) was cloned from rice cDNA with introduced *Xba*I and *Xho*I sites and inserted into pUC-YFP. The primer sequences with corresponding enzyme sites underlined are as follows: *OsTRX m2*, 5' TCTAGACGTCCCCGTCTCTCGATCG 3' and 5' CTCGAGCCTCTCGACAAATTTCTC 3'; *OsTRX m5*, 5' GCTTCTAGAATGGCGTTGGAGACGT 3' and 5' CATCTCGAGGCTGCTGACGTACTTG 3'; *BAS1*, 5' TCTAGAATGGCCGCCTGCTGCTCCT 3' and 5' CTCGAGGATGGCCGCGAAGTACTCC 3'; *OsGLK1*, 5' TCTAGAGAGATGCTTGCCGTGTCGC 3' and 5' ACTAGTTCCACACGCTGGAGGAACG 3'; *OsRpl6-2*, 5' GCTCTAGAATGGAAGCCAAGTTTTTC 3' and 5' ATGCTCGAGGGGTTTAAAGCAGAAGAC 3'. Plasmids pUC-SPYNE and pUC-SPYCE were described previously [30].

Plasmids pUC-bZIP63-YN and pUC-bZIP63-YC were made by cloning the *bZIP63 *fragment without the stop codon from *Arabidopsis *cDNA and inserting into the pUC-SPYNE and pUC-SPYCE multiple cloning sites (MCS) with *BamH*I and *Xho*I [30]. Plasmids pUC-BAS1-YN, pUC-OsTRX m2-YC and pUC-OsTRX m5-YC were constructed by transferring their coding sequences into pUC-SPYNE and pUC-SPYCE MCS at the *Xba*I and *Xho*I sites, respectively.

Plasmid DNA was prepared by standard kits, such as Omega and TIANGEN (Beijing) according to the manufacturer's instructions with some modifications. Two or more columns, each for 5-8 mL bacterial cells cultured for 12-16 h, were used for plasmid DNA purification. After the precipitated DNA was bound to the columns, the deproteinization buffer was added to remove unwanted metabolites and repeated twice. The DNA was then washed twice with wash buffer. An appropriate amount of sterilized distilled water, generally 50 μL per two columns, was added to the center of one column to elute the DNA. The DNA solution was collected by centrifugation for 1 min and then added to another column. The combined plasmid DNA from all columns was collected in one Eppendorf tube. The DNA concentration and quality were determined using a DU^® ^730 Beckman Nucleic Acid/Protein Analyzer. The DNA purified from two columns usually resulted in a concentration of 1-2 μg μL^-1^. All plasmids were stored at -20°C before use.

### Protoplast transfection

PEG-mediated transfections were carried out as described [8]. Briefly, for each sample 5-10 μg of plasmid DNA were mixed with 100 μL protoplasts (about 2 × 10^5 ^cells). For BiFC or other co-expression assays, the total plasmid DNA was between 10 μg and 15 μg. 110 μl freshly prepared PEG solution [40% (W/V) PEG 4000; Fluka, 0.2 M mannitol and 0.1 M CaCl_2_] were added, and the mixture was incubated at room temperature for 10-20 min in the dark. After incubation, 440 μL W5 solution were added slowly. The resulting solution was mixed well by gently inverting the tube, and the protoplasts were pelleted by centrifugation at 1,500 rpm for 3 min. The protoplasts were resuspended gently in 1 mL WI solution (0.5 M mannitol, 20 mM KCl and 4 mM MES at pH 5.7). Finally, the protoplasts were transferred into multi-well plates and cultured under light or dark at room temperature for 6-16 h. The amount of DNA, protoplasts and other solutions used in this transfection sysem could be scaled up or down based on experimental purposes.

### Confocal laser scanning microscopy

Protoplasts were observed using a confocal laser scanning microscope (Leica TCS 5 SP5 AOBS) and visualized by a Leica Microsystem LAS AF. GFP, YFP and mCherry were excitated at 488 nm, 514 nm and 561 nm wavelengths, respectively. The emission filters were 500-530 nm for GFP, 530-560 nm for YFP and 580-620 nm for mCherry. Chlorophyll autofluorescence was monitored using either 488 nm or 514 nm excitation wavelengths, and 650-750 nm detection windows. All fluorescence experiments were repeated independently at least three times.

### Total protein extraction, Western blot and Luciferase activity measurement

Protoplasts were harvested by centrifugation at 1,500 rpm for 3 min. Total protein was extracted with protein extraction buffer (50 mM Tris-HCl at pH 7.5, 150 mM NaCl, 5 mM EDTA, 0.2% NP-40, 0.1% Triton X-100 and Complete protease inhibitor cocktail, Roche), usually 200-300 μL for 1 mL protoplasts (approximately 2 × 10^6 ^cells). The extracts were then centrifuged at 16,000 rpm for 15 min at 4°C, and the supernatants were collected for Western blot analysis. About 20 μg of total protein per sample, determined by the Bradford Assay (BioRad), were analyzed by SDS-PAGE. Western analysis was performed with a monoclonal mouse anti-c-myc (Roche) or monoclonal rabbit anti-GFP (Abmart) primary antibody according to standard protocols [46]. Firefly luciferase and renilla luciferase activities were measured using the Dual-Luciferase^® ^Reporter Assay System (Promega). RNL [47] was co-transfected in all FLC experiments, serving as an internal control, and firefly luciferase activity was normalized to RNL activity.

### Chlorophyll fluorescence measurements

The photosynthetic properties of rice protoplasts were measured in 96-well white polystyrene plates (Corning) using an IMAGING-PAM chlorophyll fluorometer (MAXI Version; Walz, Efeltrich, Germany) with imaging areas up to 10 cm×13 cm. Areas of interest (AOI, diameter 0.5 cm) were selected randomly to record data. Images of the chlorophyll fluorescence parameters were taken under saturation pulse mode, and the concrete features were as follows: dark adaptation was 10 min for each protoplast sample; measured light intensity was 0.5 μmol m^-2 ^s^-1^; saturation pulse light was 2,700 μmol m^-2 ^s^-1 ^(duration 0.8 s; interval 20 s) and actinic light intensity was 35 μmol m^-2 ^s^-1^. Fluorescence data and the corresponding images were recorded simultaneously.

### RNA extraction and Quantitative real-time PCR analysis

Total RNA of rice protoplasts was extracted using the Omega Plant RNA kit according to the manufacturer's instructions. cDNA was made using the PrimeScript RT reagent Kit with gDNA eraser (Taraka) with 2 μg of total RNA as the template. Quantitative real-time PCR was performed with a Bio-Rad IQ5 system using SYBR to monitor double-stranded DNA products. The gene-specific primers were as follows: *OsLhcb1 *(Os09g0346500), 5' GGAAGATGGGTTTAGTGCG 3' and 5' GCTAATCAGAATAACACCACGG 3'; *OsLhcp *(Os01g0600900), 5' TACGAGTATTGGAGAGAGG 3' and 5' TAAGTAGCACGCAGGATT 3'; *GADPH *(Os03g0129300), 5' GTGGCCAACATTATCAGCAA 3' and 5' GGTCATGGTTCCCTTTACGA 3'; *RbcS *(Os12g0292400), 5' CCCGGATACTATGACGGTAGG 3' and 5' AACGAAGGCATCAGGGTATG 3'; *β-actin *(internal control), 5' CCTGACGGAGCGTGGTTAC 3' and 5' CCAGGGCGATGTAGGAAAGC 3'.

## Competing interests

The authors declare that they have no competing interests.

## Authors' contributions

YZ, JS and HW designed the study. YZ and JS optimized the protoplast system and drafted the manuscript. SD performed the FDA staining and chlorophyll fluorescence measurements. YA, JL and PW conducted the statistical analysis and FLC assays. JD and YL performed the Western blot and quantitative real-time PCR assays. BL and DF prepared materials, including rice seedlings and vectors, and provided assistance in the revision of the manuscript. HW and JW supervised the study and critically revised the manuscript. All authors read and approved the final manuscript.

## Supplementary Material

Additional file 1 **Eight-day-old rice seedlings**. Sterilized rice seeds were germinated and cultured on 1/2 MS medium with a photoperiod of 12 h light (about 150 μmol m^-2 ^s^-1^) and 12 h dark at 26 °C. Scale bar = 1 cm. Click here for file

Additional file 2 **Viability of rice green tissue protoplasts**. Rice green tissue protoplasts were stained with 0.01% fluorescein diacetate (FDA). The viable cells were visualized under a fluorescent microscope indicated by green fluorescence. A bright field image of protoplasts is also shown. Scale bar = 50 μm. Click here for file

Additional file 3 **List of recombinant plasmids used in this study**. The information of recombinant plasmids used in this study is listed. It includes transfection controls, BiFC and FLC controls, and rice and *Arabidopsis *organelle markers. Click here for file

Additional file 4 **Mis-localization of *Arabidopsis *organelle markers in heterologous rice expression system**. Transient expression of *Arabidopsis *organelle markers in rice green tissue protoplasts showed partial ambiguous localizations. CD3-958 formed rings around the chloroplasts that did not coincide with the endoplasmic reticulum (ER). CD3-1006 did not label the plasma membrane (PM) as expected but instead was found in the cytosol and nucleus. Individual and merged images of YFP and chlorophyll autofluorescence (Chl) as well as bright field images of protoplasts are shown. Scale bars = 10 μm. Click here for file
