# Supplementary figures and images for: A highly efficient rice green tissue protoplast system for transient gene expression and studying light/chloroplast-related processes
Source: Plant Methods. 2011 Sep 30;7:30. doi: 10.1186/1746-4811-7-30 (PMC3203094; doi:10.1186/1746-4811-7-30)

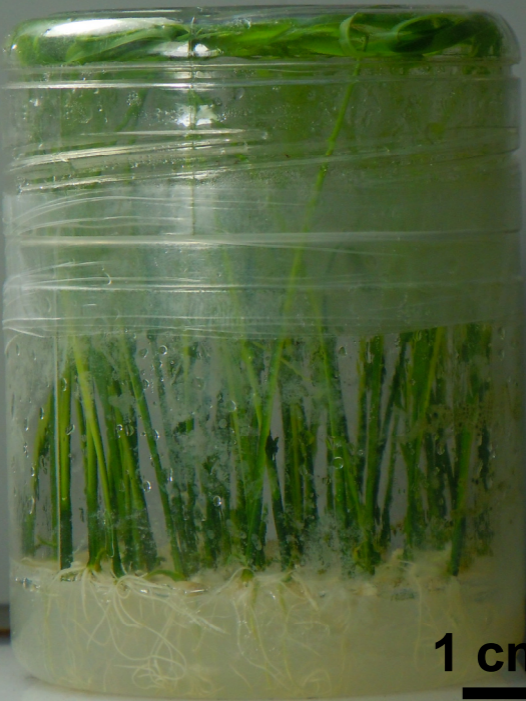

1 cm

Supplement: Additional file 1 — Eight-day-old rice seedlings. Sterilized rice seeds were germinated and cultured on 1/2 MS medium with a photoperiod of 12 h light (about 150 μmol m-2 s-1) and 12 h dark at 26 °C. Scale bar = 1 cm. [file 1746-4811-7-30-S1.PDF]

# Viable cells

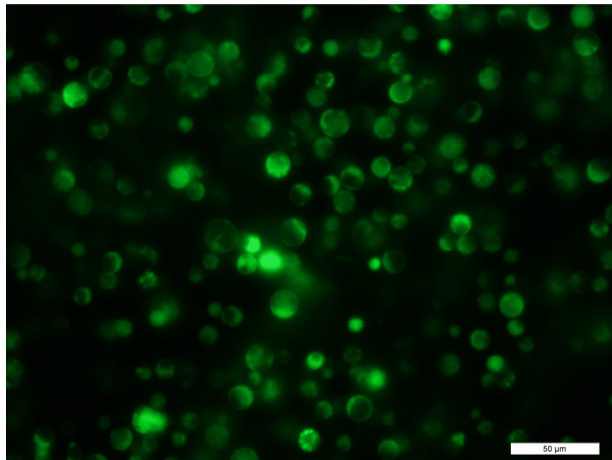

# Bright

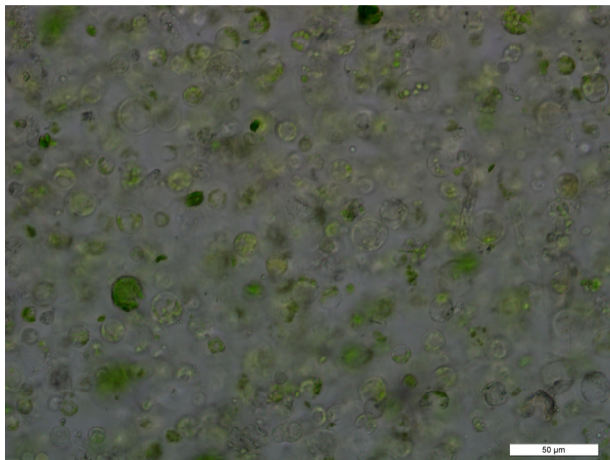

Supplement: Additional file 2 — Viability of rice green tissue protoplasts. Rice green tissue protoplasts were stained with 0.01% fluorescein diacetate (FDA). The viable cells were visualized under a fluorescent microscope indicated by green fluorescence. A bright field image of protoplasts is also shown. Scale bar = 50 μm. [file 1746-4811-7-30-S2.PDF]

**YFP**

**Chl**

**Merged**

**Bright**

**CD3-958  
(ER)**

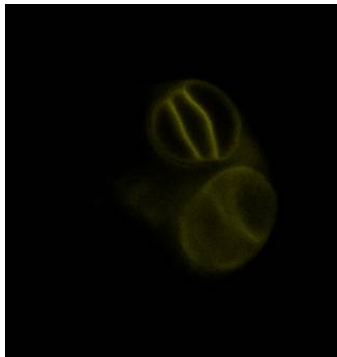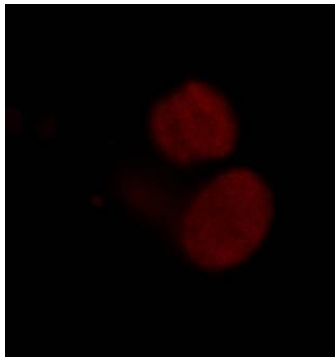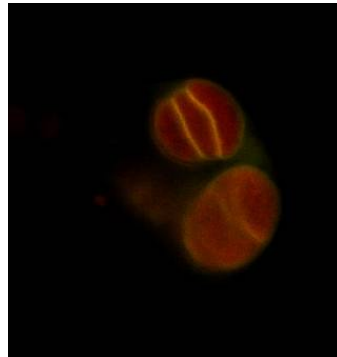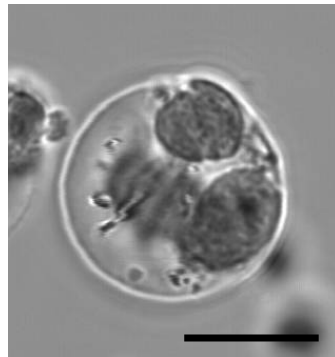

**CD3-1006  
(PM)**

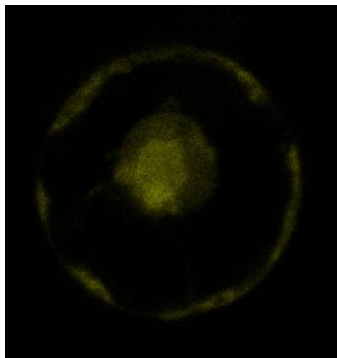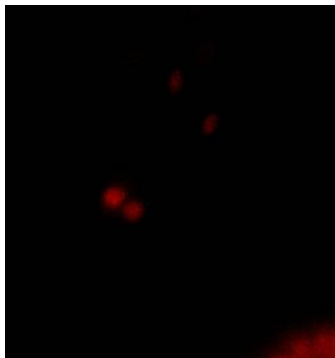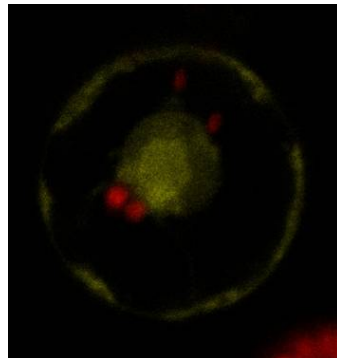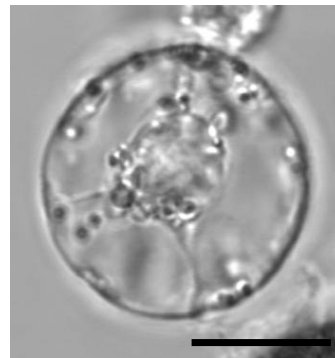

Supplement: Additional file 4 — Mis-localization of Arabidopsis organelle markers in heterologous rice expression system. Transient expression of Arabidopsis organelle markers in rice green tissue protoplasts showed partial ambiguous localizations. CD3-958 formed rings around the chloroplasts that did not coincide with the endoplasmic reticulum (ER). CD3-1006 did not label the plasma membrane (PM) as expected but instead was found in the cytosol and nucleus. Individual and merged images of YFP and chlorophyll autofluorescence (Chl) as well as bright field images of protoplasts are shown. Scale bars = 10 μm. [file 1746-4811-7-30-S4.PDF]
