# Supplementary material for: A highly efficient rice green tissue protoplast system for transient gene expression and studying light/chloroplast-related processes
Source: Plant Methods. 2011 Sep 30;7:30. doi: 10.1186/1746-4811-7-30 (PMC3203094; doi:10.1186/1746-4811-7-30)
Supplement: Additional file 3 — List of recombinant plasmids used in this study. The information of recombinant plasmids used in this study is listed. It includes transfection controls, BiFC and FLC controls, and rice and Arabidopsis organelle markers. [file 1746-4811-7-30-S3.DOC]

**Additional file 3. List of recombinant plasmids used in this study.**

| Name | Size (kb) | Resistance | Use | References |
| --- | --- | --- | --- | --- |
| pUC-GFP | ～4.5 | Ampicillin | Transfection control | This study |
| CD3-958* | ～13 | Kanamycin | ER-YFP marker |  |
| CD3-966* | ～13 | Kanamycin | Golgi-YFP marker |  |
| CD3-982* | ～13 | Kanamycin | Peroxisome-YFP marker |  |
| CD3-998* | ～13 | Kanamycin | Plastid-YFP marker |  |
| CD3-1000* | ～13 | Kanamycin | Plastid-Cherry marker |  |
| CD3-1006* | ～13 | Kanamycin | PM-YFP marker |  |
| OsTRX m2-GFP | ～5 | Ampicillin | Rice thioredoxin | This study |
| OsTRX m5-GFP | ～5 | Ampicillin | Rice Plastid marker |  |
| BAS1-GFP | ～4.3 | Ampicillin | Rice 2-Cys peroxiredoxin | This study |
| OsRpl6-2-YFP | ～4.7 | Ampicillin | Rice Mitochondrion marker |  |
| pUC- SPYNE | ～3.5 | Ampicillin | BiFC assay and Western blot |  |
| pUC- SPYCE | ～3.5 | Ampicillin | BiFC assay |  |
| pUC-bZIP63-YN | ～4.5 | Ampicillin | BiFC assay and Western blot |  |
| pUC-bZIP63-YC | ～4.5 | Ampicillin | BiFC assay |  |
| pUC-TRX m2-YC | ～4 | Ampicillin | BiFC assay | This study |
| pUC-TRX m5-YC | ～4 | Ampicillin | BiFC assay | This study |
| pUC-BAS1-YN | ～4.3 | Ampicillin | BiFC assay | This study |
| 35S::NLuc | ～5 | Ampicillin | FLC assay |  |
| 35S::CLuc | ～5 | Ampicillin | FLC assay |  |
| SGT1a-NLuc | ～5 | Ampicillin | FLC assay |  |
| CLuc-RAR1 | ～5 | Ampicillin | FLC assay |  |
| RNL | ～5 | Ampicillin | FLC assay |  |
| OsGLK1-GFP | ～5.9 | Ampicillin | Light/chloroplast-related study |  |

*Arabidopsis Biological Resource Center (ABRC, <http://abrc.osu.edu/>) stock numbers. ER: endoplasmic reticulum; PM: plasma membrane; BiFC, bimolecular fluorescence complementation; FLC, firefly luciferase complementation; RNL, renilla luciferase.
